# Development of a machine learning model to identify individuals with VCTE-derived at-risk MASH in a Spanish Mediterranean region

**DOI:** 10.3389/fdgth.2026.1829881

**Published:** 2026-09-22

**Authors:** Marta Cedenilla, David Marti-Aguado, Josep Redon, Inma Sauri-Ferrer, Jorge Navarro, Desamparados Escudero, Anton Gomez, Luis Cea-Calvo, Carlos J. Martos-Rodriguez, Javier Díaz Carnicero

**Affiliations:** 1 Value & Implementation, Global Medical & Scientific Affairs, Merck Sharp & Dohme Spain, Madrid, Spain; 2 Data Science Platform Biomedical Research Institute, Valencia, Spain; 3 Digestive Disease Department, Clinic University Hospital of Valencia, Valencia, Spain

**Keywords:** artificial intelligence, machine learning, metabolic dysfunction-associated steatohepatitis, metabolic dysfunction–associated steatotic liver disease, non-invasive test

## Abstract

**Introduction:**

While current guidelines recommend screening for liver disease in target populations, existing non-invasive tests have limitations in identifying at-risk metabolic dysfunction-associated steatohepatitis (MASH).

**Materials and methods:**

This retrospective study used two large datasets from the general population in the Valencian Community region (Spain). The primary goal was to develop a machine learning model to identify individuals with at-risk MASH. The model was constructed using a well-characterized dataset (*n* = 2,267) with available vibration-controlled transient elastography (VCTE). Supervised machine learning models (Random Forest and XGBoost) were trained using a 10-fold cross-validation to classify at-risk MASH individuals, defined as those with a controlled attenuation parameter (CAP) ≥ 275 dB/m together with a liver stiffness measurement (LSM) > 8 kPa and/or FibroScan-AST score (FAST) > 0.35. The final model, named VARM-7 (Valencia At-Risk MASH), comprised age and six commonly available laboratory variables. VARM-7 was sequentially applied to the Valencian public healthcare database (*n* = 3,411,069) to estimate the proportion of at-risk MASH individuals in the overall population, and in target populations with type 2 diabetes mellitus (T2DM) and obesity.

**Results:**

In the independent validation dataset, VARM-7 exhibited a significantly higher AUROC compared with Fibrosis-4 (FIB-4) for identifying at-risk MASH (0.84 *vs*. 0.64, respectively, *p* < 0.001). When sequentially applied to the overall Valencian Community population, 11.7% of individuals were estimated to have at-risk MASH, with increasing estimates up to 29.4% and 44.0% in subjects with obesity and T2DM, respectively.

**Conclusion:**

VARM-7 showed strong performance in identifying individuals with a VCTE-derived at-risk MASH. Our model could improve disease screening and referral pathways, but external validation and prognostic evaluation are needed before its implementation.

## 1 Introduction

Metabolic dysfunction-associated steatotic liver disease (MASLD) is the most common cause of chronic liver disease worldwide (1). Key risk factors that define this condition include obesity, type 2 diabetes mellitus (T2DM), and metabolic syndrome (2, 3). MASLD represents a continuum that ranges from simple hepatic steatosis to metabolic dysfunction-associated steatohepatitis (MASH) (4). MASH can progress to liver fibrosis, cirrhosis, and hepatocellular carcinoma. It is also a risk factor for cardiovascular disease, which is the leading cause of mortality in this population (5–7).

The prevalence of MASLD is on the rise, in parallel with the increasing epidemic of obesity and T2DM, together with the improved life expectancy of patients with cardiovascular conditions (3). Between 1990 and 2006 and 2016–2019, MASLD prevalence rose from 25.3% to 38% in the adult general population, highlighting the growing burden of the disease (8, 9). The estimated prevalence of MASH was 5.3% in 2023 and is also rising (9, 10). Despite this increasing trend, MASLD and MASH prevalences may still be underestimated due to challenges in diagnosis and low disease awareness (7, 11). Additionally, prevalence rates show substantial regional variations associated with demographic, socio-economic, and genetic factors (12–14).

While liver biopsy is the gold standard for diagnosing MASLD and MASH, it is an invasive and costly procedure that carries potential complications for patients, and is impractical for population screening and long-term monitoring (15). Consequently, liver biopsies are primarily reserved for symptomatic patients with suspected advanced disease, with the consequent overlook of many asymptomatic patients (16). MRI-based elastography is considered the non-invasive reference standard for routine quantification of hepatic fibrosis in clinical practice (17). Quantitative CT methods for staging fibrosis have demonstrated comparable performance to elastography (18). Furthermore, non-invasive tests like the Fibrosis-4 (FIB-4) index are recommended for fibrosis risk assessment in current guidelines (19–22). However, non-invasive tests also have limitations, including inconsistent predictive accuracy, moderate sensitivity for advanced fibrosis stages, and poor diagnostic performance in younger populations or in individuals with T2DM (23, 24). Additionally, many non-invasive tests lack specificity for diagnosing MASH and rely on clinical parameters that are not routinely collected in clinical practice (25, 26). Given these limitations, there is an unmet need for more accurate, non-invasive screening strategies to identify individuals at risk of MASH and improve referral pathways.

To overcome these limitations, artificial intelligence (AI) is a promising tool in medicine, increasingly used in hepatology (27). Machine learning (ML), a subfield of AI, focuses on developing predictive algorithms based on datasets. Unlike traditional statistical modeling, ML can recognize complex data patterns, which is particularly useful for identifying at-risk populations and minimizing the need for invasive liver biopsies (28, 29). In this study, we aimed to develop a ML model to identify individuals at risk of MASH among patients with T2DM and/or obesity, a population for whom current guidelines recommend screening for liver disease (21, 30). Using a large, well-characterized dataset with available vibration-controlled transient elastography (VCTE) data, we sought to refine FIB-4–based referral pathways and optimize liver disease evaluation through AI-based tools, by identifying individuals who may benefit from further non-invasive assessment or specialist referral. We also aimed to estimate the proportion of individuals at risk of MASH within this target population in the Valencian Community region of Spain.

## 2 Methods

### 2.1 Study design

A retrospective study was performed using two large datasets from the general population in Valencia, Spain. The primary objective of the study was to develop a ML model based on universally accessible variables to identify individuals at risk of MASH. The secondary objective was to estimate the proportion of individuals with at-risk MASH by applying the developed ML model in more than 3 million individuals from the Valencian Community region of Spain.

To classify at-risk MASH, different ML models were developed and applied sequentially: first to identify individuals at risk of MASLD and then to classify those at risk of MASH within this population. As the primary objective of the study was to identify the population at risk of MASH, detailed information on MASLD models is provided as Supplementary Information.

This study was conducted in accordance with the Declaration of Helsinki, following approval by the Ethics Committee of the University Hospital of Valencia (protocol code: 2022/174; May 27, 2022). In alignment with the Ethics Committee, the requirement for informed consent was waived due to the retrospective design of the study and the use of anonymized data. The study was reported in accordance with the relevant items of the TRIPOD + AI guidance for transparent reporting of prediction models using AI and ML in healthcare (31).

### 2.2 Data sources

The study included two well-characterized datasets from the general population (Figure 1). The first dataset, comprising data from 2,267 individuals from the general population in Valencia, was used to develop the ML models. This cohort belongs to a population-based nested study (ETHON dataset, NCT02749864; registry: PREVHEP-2015-01), which prospectively recruited individuals registered in the Public Health System using two-stage conglomerate sampling from 2015 to 2017 (32, 33). All individuals in this cohort underwent a VCTE exam (FibroScan® Echosens, Paris, France) after fasting for at least 3 h to quantify the controlled attenuation parameter (CAP) and liver stiffness measurement (LSM).

**Figure 1:**
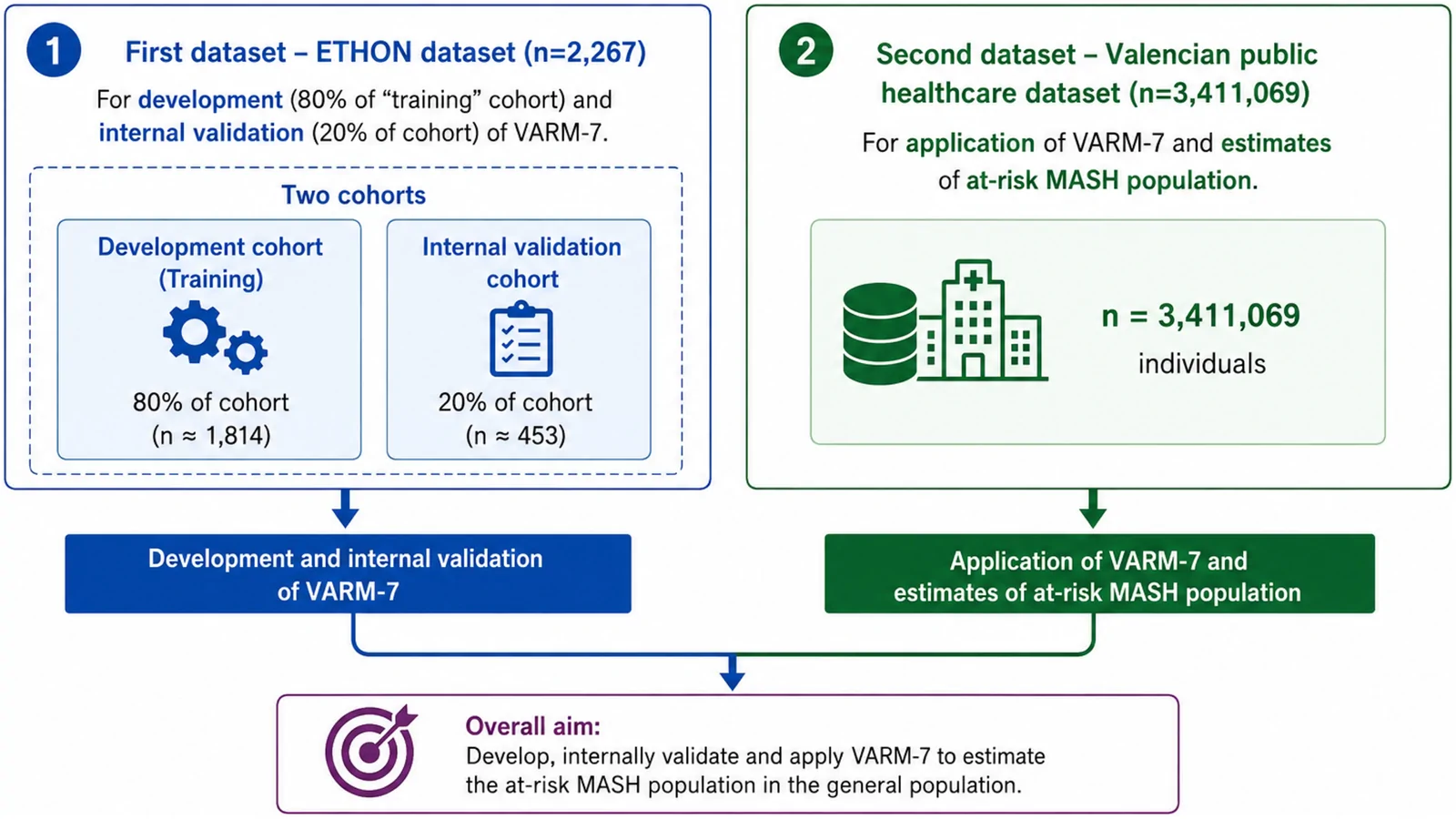
Study design and datasets.

The second dataset included data from the Valencian public healthcare database, comprising 3,411,069 individuals in 2019, with demographic, socio-economic, and clinical healthcare information across primary care, hospital care, pharmacy, and laboratory data (11). This dataset was used to estimate the proportion of individuals with a VCTE-derived at-risk MASH phenotype using the developed ML models. To this end, ML models were applied to data from adults aged 24 years or older from this public database, excluding those cases diagnosed with viral hepatitis, alcoholic hepatitis, toxic hepatitis, and autoimmune hepatitis, or with diabetes in the context of pregnancy or other situations. Adults aged ≥24 years were included based on the age range available in the database and given that FIB-4 has a lower diagnostic accuracy in younger patients (34). The proportion of individuals with a VCTE-derived at-risk MASH phenotype was estimated using ML models in the overall population and in individuals with T2DM or obesity. At-risk MASH was selected as the outcome of interest because it identifies patients who may be candidates for treatment with FDA-approved therapies for MASH.

### 2.3 Statistical analysis

Statistical analyses were conducted using the R software (version 3.6.1). The proportion of individuals predicted to have a VCTE-derived at-risk MASH phenotype was estimated by sequentially applying the developed ML models to the Valencian public healthcare database in 2019. The diagnostic performance of the ML model was compared to FIB-4. Healthcare resource use was evaluated by retrieving the following variables from the database: primary care visits, pharmacy electronic prescriptions, hospital outpatient visits, major ambulatory surgery, hospitalizations, and emergency visits, as previously described (11).

### 2.4 Machine learning model development

Supervised ML model development comprised the following steps: data quality assessment and preprocessing, training cohort division, feature selection, training, validation, and calibration. MASH cases used for building the training cohort were defined as individuals with a CAP ≥ 275 dB/m and with an LSM > 8 KPa and/or FAST score >0.35 (26, 35). The first dataset (*n* = 2,267) was randomly divided into a training cohort comprising 80% of participants and an independent hold-out validation cohort comprising 20% of participants (Figure 1). Within the training cohort, 10-fold cross-validation was used for feature selection, hyperparameter tuning and model selection. The independent hold-out validation subset was not used during feature selection, preprocessing decisions, hyperparameter tuning, undersampling, or model calibration, and was reserved for the final evaluation of model performance.

Within the training phase, two supervised ML models (Random Forest and XGBoost) were fit to the training set with a k-fold cross-validation method with k = 10 for hyperparameter optimization. The evaluated hyperparameters included the number of trees, number of prediction variables and minimum and maximum number of terminal nodes. Imbalanced data were processed using the undersampling method, which reduced the training population by eliminating samples from the majority class until an equilibrium with the minority class was achieved. Model calibration was performed by evaluating the trade-off between evaluation metrics, with a particular focus on sensitivity. The proposed calibration threshold was the inflection point among the metrics that maximized the model's utility. The predictive performance of calibrated models was evaluated on the validation set through sensitivity, specificity, positive predictive value, negative predictive value, and the area under the receiver-operating characteristic curve (AUROC). The AUROC of the ML model was compared to the AUROC of FIB-4 using DeLong test.

Features assessed comprised 33 clinical variables (Supplementary Table S1). Feature selection was based on two criteria: importance and opportunity. Feature importance involved eliminating variables with an importance score lower than 0.1 (on a normalized scale of 1). The process was repeated with new sets of models to refine the selection. Selection based on opportunity considered clinical variables that had limited availability of results. Feature selection was based on the number of individuals with available data, with features being progressively added until the metrics showed only marginal improvement compared to the reduction in the target model population. Feature selection was performed using two complementary criteria: predictive contribution and practical availability in routine care. Candidate predictors were first ranked according to model-derived variable importance within the training workflow. Variables with normalized importance below 0.10 were excluded. Candidate models were then refitted using progressively smaller sets of variables, and the final set was selected when additional variables produced only limited improvement in validation performance relative to their impact on data availability. This threshold used for the final stopping decision was based on a comparative assessment of the performance metrics, with particular emphasis on the performance plateau of the AUROC and prioritizing sensitivity. The diagnostic performance metrics of FIB-4 was calculated using age-adjusted cutoffs (>1.3 if <65 years and >2.0 if ≥65 years) (21).

Patients with missing data were not included in the model training. We chose not to impute the missing variables due to the proportion of patients who would be affected, in order to avoid introducing *post hoc* biases resulting from data handling. To assess the potential for bias, the distribution of patients with missing values was examined in relation to the available data, and no evidence of systematic bias due to this cause was found.

## 3 Results

### 3.1 Machine learning model development

Supervised ML models were developed using the first dataset with 2,267 participants: 1,814 (80%) comprised the training cohort and 453 (20%) the validation cohort. The demographic and clinical characteristics of the overall dataset are presented in Table 1. Both cohorts showed comparable demographic and clinical characteristics.

**Table 1:** Demographic and clinical characteristics of the dataset used for machine learning model development.

Variables	First dataset including training and validation cohort (*n* = 2,267)
Age (years)	49.2 (13.4)
BMI (kg/m^2^)	26.8 (5.0)
Waist circumference (cm)	92.1 (13.5)
Hemoglobin (g/dL)	14.2 (1.4)
MCV (fL)	90.7 (5.0)
Leucocytes (×10^9^ /L)	6.8 (1.9)
Neutrophils (×10^9^ /L)	3.8 (1.3)
Lymphocytes (×10^9^ /L)	2.3 (0.7)
Platelets (×10^9^ /L)	249.7 (62.2)
Glucose (mg/dL)	100.0 (25.5)
Total cholesterol (mg/dL)	205.4 (40.7)
LDL cholesterol (mg/dL)	132.9 (32.0)
HDL cholesterol (mg/dL)	57.3 (13.3)
Triglycerides (mg/dL)	115.0 (75.7)
AST (U/L)	24.6 (10.1)
ALT (U/L)	24.8 (16.0)
GGT (U/L)	33.9 (49.3)
Bilirubin (mg/dL)	0.6 (0.4)
LDH (U/L)	348.2 (69.4)
Albumin (g/dL)	4.5 (0.3)
Ferritin (ng/mL)	123.4 (182.8)

Data are expressed as mean (SD).

ALT, alanine aminotransferase; AST, aspartate aminotransferase; BMI, body mass index; GGT, gamma-glutamyltransferase; HDL, high-density lipoprotein; LDH, lactate dehydrogenase; LDL, low-density lipoprotein; MCV, mean corpuscular volume.

The final MASH model, named VARM-7 (Valencia at-risk MASH), was a Random Forest classifier. As XGBoost did not improve model performance across the evaluated metrics, the Random Forest approach was selected given its relative simplicity and greater interpretability. VARM-7 used seven variables: age, platelets, glucose, cholesterol, triglycerides, alanine aminotransferase, and creatinine. Variable importance of VARM-7 is shown in Table 2. In the independent validation cohort, FIB-4 showed an AUROC of 0.64, sensitivity of 0.68, and a specificity of 0.61. In comparison, VARM-7 showed an AUROC of 0.84, a sensitivity of 0.72, and a specificity of 0.87 (Table 3; Figure 2A). Using DeLong test, VARM-7 showed a significantly higher AUROC than FIB-4 (*p* < 0.001) for the identification of at-risk MASH patients. Variable importance and performance metrics of MASLD models are shown in Supplementary Tables S2, S3. Calibration metrics are shown in Figure 2B.

**Table 2:** Variable importance of VARM-7 based on the random forest classifier.

Variable	Rank	Importance (%)
Alanine aminotransferase	1	100.0
Glucose	2	40.4
Cholesterol	3	14.3
Platelets	4	13.5
Age	5	13.0
Creatinine	6	11.6
Triglycerides	7	10.2

**Table 3:** Performance metrics for the VARM-7 model and FIB-4 index in the validation dataset from the training cohort.

Metric	VARM-7	FIB-4
Sensitivity	0.72 (0.58–0.86)	0.68 (0.54–0.82)
Specificity	0.87 (0.83–0.92)	0.61 (0.57–0.66)
PPV	0.50 (0.38–0.63)	0.23 (0.11–0.35)
NPV	0.95 (0.92–0.98)	0.91 (0.88–0.94)
AUROC	0.84 (0.77–0.90)	0.64 (0.57–0.69)

AUROC, area under the receiver-operating characteristic curve; FIB-4, Fibrosis-4; NPV, negative predictive value; PPV, positive predictive value; VARM-7, Valencia at-risk MASH model.

The cutoff of VAMR-7 was 0.60, which was selected based on a comparative assessment of the performance metrics, with particular emphasis on the performance plateau of AUROC and prioritizing sensitivity in the calibration plot. The cutoffs for FIB-4 were age-adjusted as follows: >1.3 if <65 years and >2.0 if ≥65 years.

**Figure 2:**
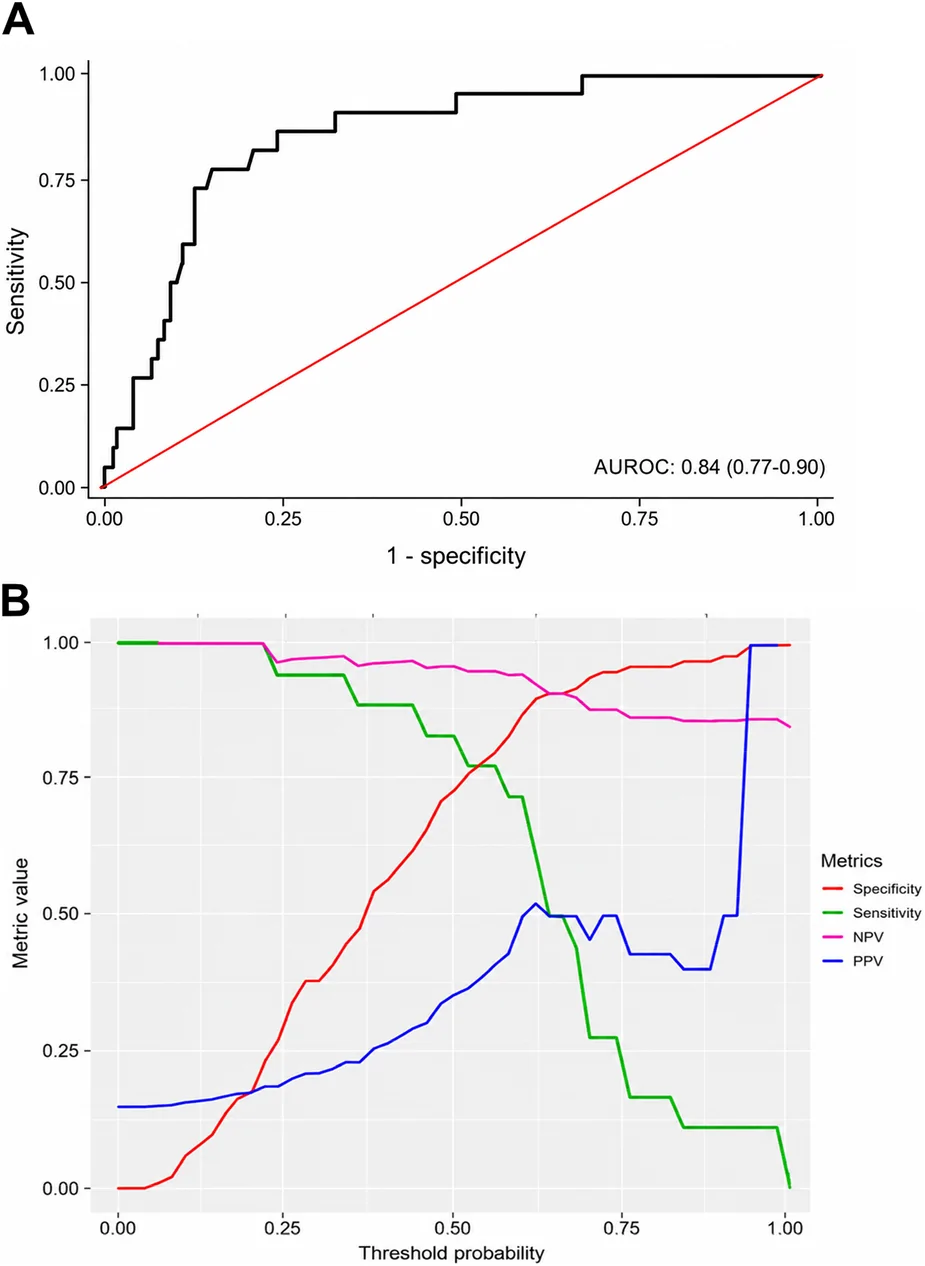
**(A)** receiver operating characteristic curve for the VARM-7 model. **(B)** Calibration plot for the VARM-7 model performance in terms of sensitivity, specificity and positive and negative predictive values by all possible cut-off values.

### 3.2 Model application to the valencian community region

Supervised ML models were sequentially applied to the second dataset from the Valencian public healthcare database, comprising 3,411,069 individuals in 2019. Within this population, 67% had all the variables required to use VARM-7. Among those classified as at risk of MASLD (*n* = 1,573,311), 398,812 (25.3%) had VCTE-derived at-risk MASH phenotype with VARM-7, representing 11.7% of the overall population. When the models were applied to individuals with obesity and T2DM, 204,077 (29.6%) and 178,465 (47.7%) of those positive for MASLD had VCTE-derived at-risk MASH phenotype. These figures correspond to an estimated 29.4% of the population with obesity and 44.0% of the population with T2DM (Figure 3). The proportion of individuals at risk of MASLD in the overall, obesity, and T2DM populations is shown in Supplementary Figure S1. The model development workflow and its estimated population-level impact are summarized in Figure 4.

**Figure 3:**
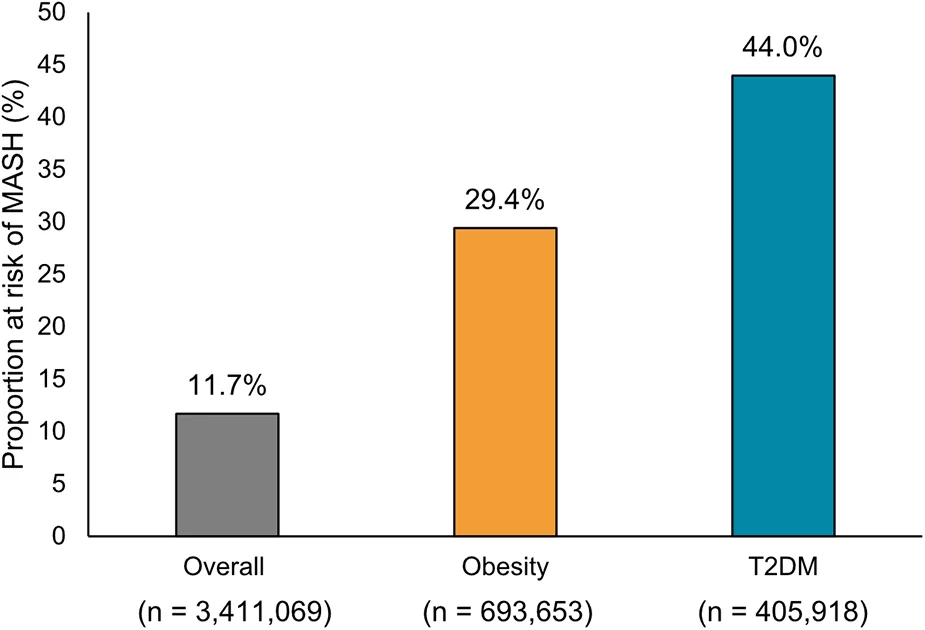
Proportion of individuals at risk of MASH in the overall population and in individuals with obesity and type 2 diabetes mellitus. The figure shows the proportion of individuals at risk, relative to 2019. MASH, metabolic dysfunction-associated steatohepatitis; T2DM, type 2 diabetes mellitus.

**Figure 4:**
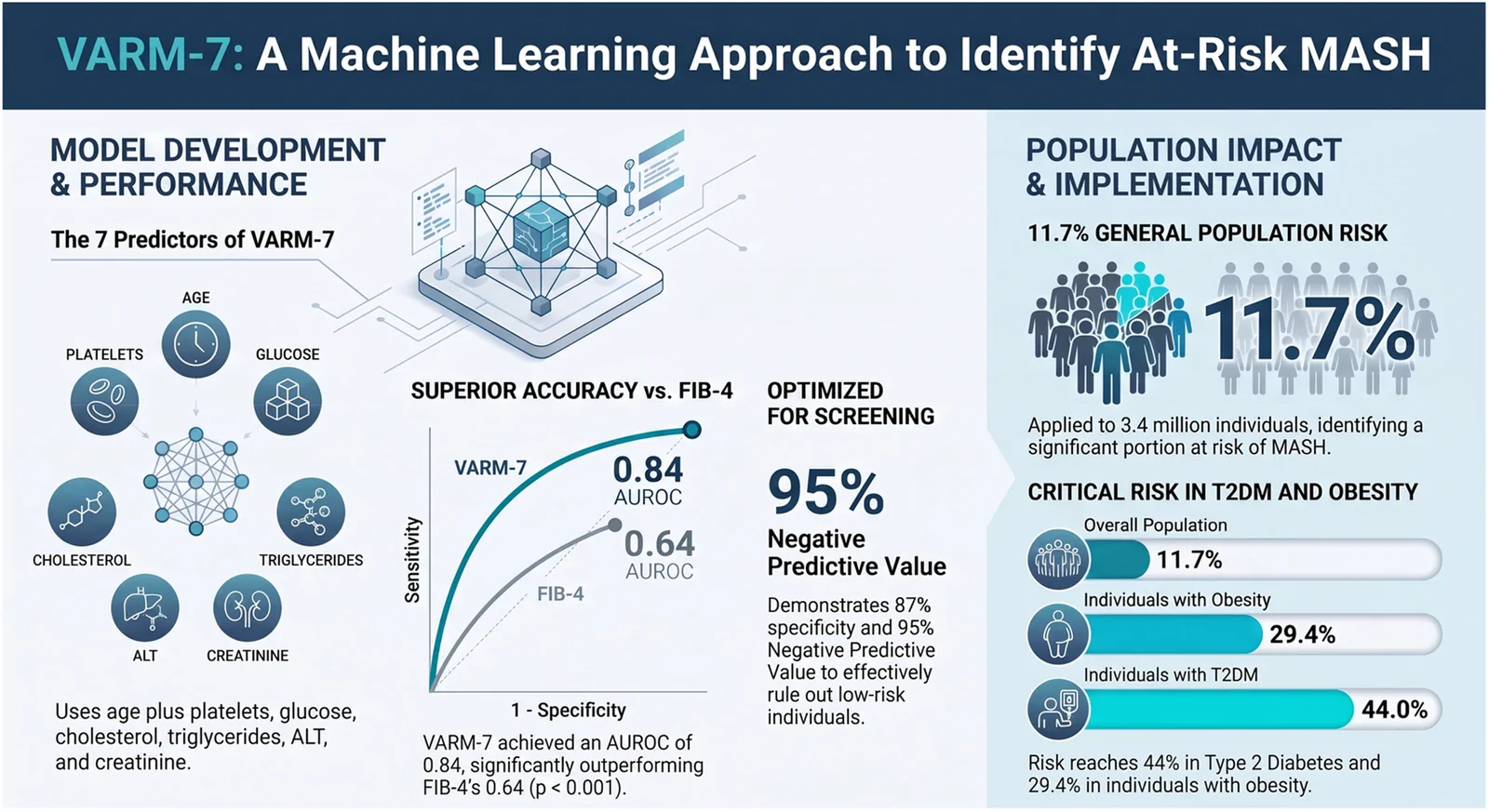
Graphical abstract with the main results of the study.

Table 4 shows the demographic and clinical characteristics of individuals at risk of MASH. Demographic and clinical characteristics of individuals at risk of MASLD are shown in Supplementary Table 4. When assessing healthcare resource utilization among individuals at risk of MASH, we observed that 97.4% attended primary care visits, while 30.7% had emergency department visits. The population with T2DM showed the highest resource use (Table 5).

**Table 4:** Demographic and clinical characteristics of individuals at risk of MASH.

Variables	At-risk MASH (*N* = 398,812)
Age (years)	65.0 (11.4)
Sex	
Men, *n* (%)	180,033 (45.1)
Weight (kg)	83.37 (17.0)
BMI (kg/m^2^)	31.4 (5.4)
Waist circumference (cm)	106.4 (11.6)
SBP (mm Hg)	132.2 (12.8)
DBP (mm Hg)	78.2 (9.5)
Diabetes duration (years)	7.5 (3.8)
HbA_1c_ (%)	6.6 (1.2)
Fasting glucose (mg/dL)	121.2 (35.3)
Total cholesterol (mg/dL)	179.7 (40.2)
HDL cholesterol (mg/dL)	50.1 (12.9)
LDL cholesterol (mg/dL)	100.7 (33.6)
Triglycerides (mg/dL)	154.1 (91.8)
Creatinine (mg/dL)	0.9 (0.3)
Albumin (g/L)	4.4 (2.7)
eGFR (mL/min/1.73 m^2^)	81.4 (17.7)
AST (U/L)	28.7 (17.3)
ALT (U/L)	31.4 (21.2)
Platelets (×10^9^ /L)	228.7 (62.4)
Uric acid (mg/dL)	5.6 (1.5)
Iron (μg/dL)	83.6 (31.5)
Ferritin (ng/mL)	147.9 (145.8)
Transferrin (mg/dL)	267.5 (50.6)
Leucocytes (×10^9^ /L)	7.4 (2.0)
CRP (nmol/L)	6.9 (18.3)
Vitamin D (ng/mL)	25.1 (12.5)

Data are expressed as mean (standard deviation, SD), unless otherwise specified.

ALT, alanine aminotransferase; AST, aspartate aminotransferase; BMI, body mass index; CRP, C-reactive protein; DBP, diastolic blood pressure; eGFR, estimated glomerular filtration rate; HbA_1c_, glycated hemoglobin; HDL, high-density lipoprotein; LDL, low-density lipoprotein; MASH, metabolic dysfunction–associated steatohepatitis; SBP, systolic blood pressure; SD, standard deviation.

**Table 5:** Annual use of healthcare resources in individuals at risk of MASH in the overall, T2DM, and obesity populations.

	Overall (*N* = 398,812)	Obesity (*N* = 204,077)	T2DM (*N* = 178,465)
At risk of MASH, %			
Primary care visits	97.4%	97.5%	98.5%
Pharmacy electronic prescription	96.2%	96.2%	98.0%
Hospital outpatient visits	71.7%	72.8%	74.3%
Major ambulatory surgery	2.3%	2.4%	2.4%
Hospitalizations	4.3%	4.7%	4.7%
Emergency visits	30.7%	32.3%	32.3%

The table shows the annual proportion of individuals who used each resource relative to 2019.

MASH, metabolic dysfunction–associated steatohepatitis; T2DM, type 2 diabetes mellitus.

## 4 Discussion

In this study, we present a novel ML model named VARM-7, based on commonly available analytical variables and age, designed to identify individuals with a VCTE-derived at-risk MASH phenotype. The developed model obtained a significantly higher AUROC than the recommended FIB-4 index for liver disease screening. Within the Valencian Community region of Spain, VARM-7 classified 11.7% of the overall population, 29.4% of individuals with obesity, and 44.0% of individuals with T2DM as meeting this surrogate-defined risk category of at-risk MASH.

Given the rising prevalence of MASLD, optimizing screening and referral pathways to identify individuals at risk of MASH is crucial (10). While liver biopsy remains the gold standard for diagnosing MASH, it is not practical for overall screening or follow-up (15). Alternative diagnostic methods, such as non-invasive scores, are increasingly used but may not fully capture the complex interplay of clinical variables and comorbidities related to MASH (19–22)*.* Furthermore, they show limited performance in certain populations like T2DM (23, 24). Current clinical practice guidelines recommend performing VCTE or initiating lifestyle interventions in patients with a FIB-4 score >1.3. However, some studies have reported that the proportion of patients exceeding this threshold is remarkably high in non-selected populations (24, 36). This high prevalence suggests that relying solely on a FIB-4 > 1.3 may overwhelm healthcare systems with unnecessary referrals or interventions. Therefore, alternative non-invasive tests are needed to identify individuals at risk of MASH and improve patient stratification and referral. To address this need, ML models provide a data-driven approach capable of identifying patterns and interactions among variables. These models can be updated and tailored to different populations, improving their applicability and generalizability (28, 29).

In this context, we developed a supervised ML model to identify individuals at risk of MASH. Unlike other models that rely on complex or difficult-to-obtain data, a key strength of VARM-7 is that it requires only seven variables that are routinely available in clinical practice. The selected predictors overlap with variables frequently incorporated in non-invasive and AI-based models for MASLD/MASH or liver fibrosis, including age, triglycerides, or alanine aminotransferase (37–42). This combination of data accessibility and model simplicity supports their feasibility across different healthcare settings.

The selected model performed satisfactorily in predicting individuals at risk of MASH, achieving an AUC of 0.84. Compared with previous models developed to identify patients with MASH based on ML methodologies, our approach shows similar (39) or higher performance (43). Likewise, a meta-analysis provided a pooled sensitivity of 0.80 and specificity of 0.69 for identifying MASH with ML models based on clinical datasets (44). In a comparative diagnostic accuracy study using data from the LITMUS meta-cohort, none of the evaluated biomarkers met the 0.80 threshold in detecting MASH and clinically significant fibrosis (45). In a recent systematic review, the AUROC values for AI models to identify MASLD ranged from 0.503 to 0.985 (41). Importantly, VARM-7 exhibited a significantly higher AUROC compared with FIB-4 in the independent validation dataset. This could be due to the ability of AI models to identify subtle patterns and complex relationships that conventional scores cannot capture (41). The performance metrics of our approach demonstrate high specificity and acceptable sensitivity, along with excellent negative predictive value and a moderate positive predictive value. This suggests that the model is suitable for ruling out individuals not at risk, although there is some potential for false positives. Within this context, the proposed model appears feasible for the target population, as it could serve as an initial screening tool that may minimize the need for unnecessary testing in low-risk individuals. Conversely, because of the possibility of false-positive results, individuals identified as at risk can be referred for further non-invasive assessments, thereby optimizing resource utilization.

When applied to individuals in the Valencian public healthcare database with complete data for all VARM-7 predictors, the model classified 11.7% of the overall population as meeting the VCTE-derived at-risk MASH phenotype. Based on ICD codes, we previously reported a prevalence of 0.04% for MASH from the same database, highlighting the underdiagnosis of this condition (11). Conversely, the proportion of individuals predicted to have and thus identified as at risk in our study is closer to that of a population-based study from various Spanish regions, showing a prevalence between 13% and 15% for MASH (32). These results highlight that despite increasing awareness of the disease, MASH is often overlooked in clinical practice, suggesting that many individuals could be at risk without receiving a coded diagnosis, as previously observed (46). However, it is important to note this should be interpreted as a model-based estimate of a surrogate-defined risk category rather than as the prevalence of biopsy-confirmed MASH. The healthcare database application was not an external validation because VCTE/FAST or biopsy-confirmed outcome data were not available in that database. Instead, this analysis shows the potential population-level implementation of VARM-7. The estimates may be affected by missingness, spectrum shift, and transportability limitations, and should therefore be confirmed in external datasets with measured outcomes.

While our study lacks external validation and prognostic confirmation for individuals at risk, we noted some characteristics that suggest our predictions are accurate. Specifically, the baseline characteristics of the population at risk of MASH were consistent with the presence of metabolic syndrome or liver disease. Similarly, the high resource utilization also aligns with individuals with an underlying condition, although this observation requires further confirmation. Additionally, the proportion of individuals at risk of MASH was 3.8 times and 2.5 times higher in populations with T2DM and obesity, respectively, compared to the overall population. This ratio closely resembles the findings based on ICD diagnoses (3.6 and 2.5), although with lower prevalence rates (11). Lastly, the higher prevalence of MASH in obesity and T2DM populations aligns with current guidelines recommending that individuals with these conditions should be screened for MASLD (21, 30). Further studies are necessary to validate our model in independent datasets and assess the applicability of this approach in real-world settings. However, the initial evidence, along with performance metrics, suggests that it is well-suited for the study population.

This study has some limitations that are worth considering. First, model development was based on a Spanish cohort with a relatively limited sample size and restricted to individuals aged 24 years or older. Nevertheless, comparable sample sizes have been employed in similar studies (37, 43, 47). Therefore, further research is needed to optimize model performance by incorporating external datasets with diverse demographics. Second, the reference outcome was based on VCTE-derived criteria rather than liver biopsy-confirmed MASH. Although this approach is aligned with the pragmatic and non-invasive purpose of the model, CAP, LSM, and FAST are surrogate measures and may result in outcome misclassification. Therefore, VARM-7 should be interpreted as identifying individuals with a surrogate-defined at-risk phenotype rather than diagnosing histological MASH. Third, external validation was not performed, and application to the Valencian public healthcare database should be considered model implementation rather than validation because measured VCTE/FAST or biopsy outcomes were unavailable. Fourth, the sequential model approach and the proportion of patients unclassified by the model could hinder its implementation in clinical practice. Specifically, 67% of individuals in the public healthcare database had complete data for all VARM-7 predictors. Since individuals with complete laboratory data may differ from those without complete data, this could potentially limit generalizability. The same limitation applies to feature selection, which could be influenced by specific characteristics of the dataset and could be validated using an external cohort. Lastly, variable importance was model- and dataset-dependent, and random undersampling may affect predicted probabilities, calibration, PPV, NPV, and model-based population estimates; therefore, calibration and recalibration are important before clinical implementation. Although VARM-7 showed good discrimination in the independent validation dataset, it was not compared with conventional multivariable logistic regression models using the same predictors. Therefore, the incremental value attributable specifically to the machine-learning algorithm remains uncertain. Future studies should conduct a prespecified head-to-head comparison using identical development and validation samples.

In conclusion, VARM-7 demonstrated high performance in identifying individuals with a VCTE-derived at-risk MASH phenotype using routinely available clinical variables. When sequentially applied to the Valencian Community database, it estimated that 11.7% of the overall population, 29.4% of the population with obesity, and 44.0% with T2DM were at risk of MASH. Our results suggest that the use of AI-based tools could aid in early and more comprehensive disease management by streamlining and accelerating current screening and referral pathways, which may be particularly beneficial for high-risk populations.

## Data Availability

The original contributions presented in the study are included in the article/Supplementary Material, further inquiries can be directed to the corresponding author.
