# Supplementary material for: Development of a machine learning model to identify individuals with VCTE-derived at-risk MASH in a Spanish Mediterranean region
Source: Front Digit Health. 2026 Sep 22;8:1829881. doi: 10.3389/fdgth.2026.1829881 (PMC13638652; doi:10.3389/fdgth.2026.1829881)
Supplement: Supplementary file 1 [file Datasheet1.docx]

Supplementary Material

# 1 Supplementary Data

The final models required a sequential approach to broaden their application across the entire population. Three models were developed: two for MASLD and one for MASH classification. The two MASLD models were built using different sets of variables to maximize their applicability across a broader population. MASLD models 1 and 2 were random forest classifiers incorporating 21 and 12 variables, respectively. MASLD 1 was derived from clinical variables, whereas MASLD 2 was based on comorbidities (**supplementary Table 1**). Model application follows a stepwise approach. First, MASLD 1 should be applied to cases with clinical variables available; alternatively, MASLD 2 should be applied to cases where MASLD 1 could not be applied. Finally, the MASH model should be applied to all cases classified as at risk of MASLD by either MASLD 1 or MASLD 2 models.

MASLD cases used for building the training dataset were defined based on transient elastography results, according to the following criteria. Individuals with a CAP ≥ 275 dB/m were identified as true **MASLD, following the** criterion defined by the 2021 EASL clinical practice guidelines (1).

For models based on clinical variables, the latest measurement available for the year was considered, while the presence or absence of comorbidities was assessed at the end of the year.

# 2 Supplementary Figures and Tables

**Supplementary Table 1.** Variables analyzed for machine learning development

**Demographic, clinical, and analytical variables**

Age

Sex

Weight

Height

BMI

Waist circumference

Hemoglobin

Hematocrit

MCV

Leukocytes

Neutrophils

Lymphocytes

Eosinophils

Platelets

INR

Glucose

Urea

Creatinine

eGFR

Total cholesterol

LDL cholesterol

HDL cholesterol

Triglycerides

AST

ALT

GGT

ALP

Total bilirubin

LDH

Sodium

Potassium

Albumin

Ferritin

**Comorbidities**

T2DM

Obesity

Dyslipidemia

Chronic Kidney Disease

Heart Failure

Gallstones

Intestinal Malabsorption

Hypertension

Metabolic Syndrome

Polycystic Ovary Syndrome

Hypothyroidism

Obstructive Sleep Apnea Syndrome

ALP, alkaline phosphatase; ALT, alanine aminotransferase; AST, aspartate aminotransferase; BMI, body mass index; eGFR, estimated glomerular filtration rate; GGT, gamma-glutamyltransferase; HDL, high-density lipoprotein; LDH, lactate dehydrogenase; LDL, low-density lipoprotein; MCV, mean corpuscular volume; T2DM, type 2 diabetes mellitus.

Supplementary Table 2. Variable importance of MASLD models based on the random forest classifier

| **Variable** | **Rank** | **Importance (%)** |
| --- | --- | --- |
| Waist circumference | 1 | 100.0 |
| BMI | 2 | 97.0 |
| Triglycerides | 3 | 40.0 |
| Age | 4 | 36.0 |
| Glucose | 5 | 34.1 |
| GGT | 6 | 30.1 |
| HDL | 7 | 29.4 |
| Lymphocytes | 8 | 29.1 |
| Ferritin | 9 | 26.7 |
| Neutrophils | 10 | 24.9 |
| LDH | 11 | 24.5 |
| Leukocytes | 12 | 22.4 |
| Platelets | 13 | 22.3 |
| Bilirubin | 14 | 21.7 |
| Cholesterol | 15 | 19.3 |
| Hemoglobin | 16 | 19.1 |
| LDL | 17 | 18.1 |
| ALT | 18 | 17.5 |
| MCV | 19 | 17.4 |
| AST | 20 | 14.9 |
| Albumin | 21 | 11.7 |

ALT, alanine aminotransferase; AST, aspartate aminotransferase; BMI, body mass index; GGT, gamma-glutamyltransferase; HDL, high-density lipoprotein; LDH, lactate dehydrogenase; LDL, low-density lipoprotein; MCV, mean corpuscular volume.

Supplementary Table 3. Performance metrics for the MASLD models

| **Metric** | **MASLD 1**  **(Cutoff: 0.57)** | **MASLD 2**  **(Cutoff: 0.58)** |
| --- | --- | --- |
| Sensitivity | 0.64 | 0.69 |
| Specificity | 0.83 | 0.76 |
| PPV | 0.65 | 0.60 |
| NPV | 0.82 | 0.83 |
| AUROC | 0.82 | 0.76 |

AUROC, area under the receiver-operating characteristic curve; MALSD, Metabolic dysfunction-associated steatotic liver disease; NPV, negative predictive value; PPV, positive predictive value.

**Supplementary Table 4.** Demographic and clinical characteristics of individuals at risk of MASLD and MASH

| **Overall population**  **(N = 3,411,069)** | | |
| --- | --- | --- |
| **Variables, mean (SD)** | **At risk of MASLD**  **(N = 1,573,311)** | **At risk of MASH**  **(N = 398,812)** |
| Age (years) | 65.1 (14.1) | 65.0 (11.4) |
| Sex |  |  |
| Men, n (%) | 736,702 (46.8) | 180,033 (45.1) |
| Weight (kg) | 79.75 (16.8) | 83.37 (17.0) |
| BMI (kg/m^2^) | 30.5 (5.5) | 31.4 (5.4) |
| Waist circumference (cm) | 104.0 (11.8) | 106.4 (11.6) |
| SBP (mm Hg) | 131.0 (13.4) | 132.2 (12.8) |
| DBP (mm Hg) | 77.8 (9.7) | 78.2 (9.5) |
| Diabetes duration (years) | 7.5 (3.9) | 7.5 (3.8) |
| HbA_1c_ (%) | 6.3 (1.1) | 6.6 (1.2) |
| Fasting glucose (mg/dL) | 105.9 (29.8) | 121.2 (35.3) |
| Total cholesterol (mg/dL) | 187.2 (39.4) | 179.7 (40.2) |
| HDL cholesterol (mg/dL) | 54.3 (14.4) | 50.1 (12.9) |
| LDL cholesterol (mg/dL) | 107.9 (33.4) | 100.7 (33.6) |
| Triglycerides (mg/dL) | 132.7 (77.1) | 154.1 (91.8) |
| Creatinine (mg/dL) | 0.9 (0.5) | 0.9 (0.3) |
| Albumin (g/L) | 25.8 (44.1) | 27.0 (44.4) |
| eGFR (ml/min/1.73 m^2^) | 77.4 (20.5) | 81.4 (17.7) |
| AST (U/L) | 23.2 (12.5) | 28.7 (17.3) |
| ALT (U/L) | 21.9 (14.7) | 31.4 (21.2) |
| Platelets (× 10^9^/L) | 237.3 (65.4) | 228.7 (62.4) |
| Uric acid (mg/dL) | 5.5 (1.5) | 5.6 (1.5) |
| Iron (μg/dL) | 80.9 (30.7) | 83.6 (31.5) |
| Ferritin (ng/mL) | 124.5 (127.9) | 147.9 (145.8) |
| Transferrin (mg/dL) | 258.3 (50.8) | 267.5 (50.6) |
| Leucocytes (× 10⁹/L) | 7.2 (2.0) | 7.4 (2.0) |
| CRP (nmol/L) | 7.0 (18.101) | 6.9 (18.3) |
| Vitamin D (ng/mL) | 26.0 (13.2) | 25.1 (12.5) |

Data are expressed as mean (standard deviation, SD) unless otherwise specified.

ALT, alanine aminotransferase; AST, aspartate aminotransferase; BMI, body mass index; CRP, C-reactive protein; DBP, diastolic blood pressure; eGFR, estimated glomerular filtration rate; HbA_1c_, glycated hemoglobin; HDL, high-density lipoprotein; LDL, low-density lipoprotein; MASLD, metabolic dysfunction-associated steatotic liver disease; MASH, metabolic dysfunction–associated steatohepatitis; SBP, systolic blood pressure; SD, standard deviation.

.

## 2.1 Supplementary Figures

**Supplementary Figure 1.** Proportion of individuals at risk of MASLD in the overall population and in individuals with obesity and type 2 diabetes mellitus.

The figure shows the proportion of individuals at risk relative to 2019.

MASLD, metabolic dysfunction-associated steatotic liver disease; T2DM, type 2 diabetes mellitus.

**References**

1.	Berzigotti A, Tsochatzis E, Boursier J, Castera L, Cazzagon N, Friedrich-Rust M, Petta S, Thiele M. EASL Clinical Practice Guidelines on non-invasive tests for evaluation of liver disease severity and prognosis – 2021 update. *J Hepatol* (2021) **75**:659–689. doi:10.1016/j.jhep.2021.05.025
